# Determinants of prepaid systems of healthcare financing: a worldwide country-level perspective

**DOI:** 10.1007/s10754-021-09301-w

**Published:** 2021-03-31

**Authors:** Andrea M. Leiter, Engelbert Theurl

**Affiliations:** 1 Faculty of Economics and Statistics, University of Innsbruck, Universitaetsstrasse 15, 6020 Innsbruck, Austria; 2 Faculty of Economics and Statistics, University of Innsbruck, Innsbruck, Austria

**Keywords:** Health care financing, Prepaid health care financing systems, Out-of-pocket payments, Panel data analysis, I13, I14, H51, C33

## Abstract

In this paper we examine determinants of prepaid modes of health care financing in a worldwide cross-country perspective. We use three different indicators to capture the role of prepaid modes in health care financing: (i) the share of total prepaid financing as percent of total current health expenditures, (ii) the share of voluntary prepaid financing as percent of total prepaid financing, and (iii) the share of compulsory health insurance as percent of total compulsory prepaid financing. In the econometric analysis, we refer to a panel data set comprising 154 countries and covering the time period 2000–2015. We apply a static as well as a dynamic panel data model. We find that the current structure of prepaid financing is significantly determined by its different forms in the past. The significant influence of GDP per capita, governmental revenues, the agricultural value added, development assistance for health, degree of urbanization and regulatory quality varies depending on the financing structure we look at. The share of the elderly and the education level are only of minor importance for explaining the variation in a country’s share of prepaid health care financing. The importance of the mentioned variables as determinants for prepaid health care financing also varies depending on the countries’ socio-economic development. From our analysis we conclude that more detailed information on indicators which reflect the distribution of individual characteristics (such as income, family size and structure and health risks) within a country’s population would be needed to gain deeper insight into the decisive determinants for prepaid health care financing.

## Introduction

“Money is the mother’s milk of health care. However, money does not automatically produce efficient, equitable, and effective health care. (…) The financing method chosen is of critical importance because it determines the risk-pooling arrangement and the distribution of the cost burden. It also places the financial decision-making power in the hands of a particular organization, which will decide resource allocation and distribution of services and will choose a payment method to provide incentives to providers.” (Hsiao, 2007, 950). With this statement Hsiao (2007) starts his plea for a broader and more systematic view of health care financing. He argues that financing is not only an instrument to raise resources for the production of health care services. Different modes of health care financing need specific forms of organizing the financing process (including the stages of collecting, pooling, purchasing) and offer diverse incentives for an effective, efficient and equitable coverage of health care risks, health care provision and health care utilization.

In this paper we focus on examining the spread and development of prepaid health care financing. The role of prepaid health care financing—generated by governmental revenues, compulsory health insurance contributions or private health insurance premiums—compared to individual out of pocket (in the following OOP) payments in the case of health care service utilization is a crucial trade-off in this respect. Individually, poor health and as a consequence health expenditure are to a large extent unpredictable and reduce the possibilities to consume other necessities and amenities of life. If individuals are risk averse, they want to smooth their consumption path and therefore demand prepaid health care financing schemes with their risk-pooling characteristics. Consequently, the implementation of prepaid systems of health care financing seems to have high potential for a Pareto improvement. This is especially true if direct payments are substantial and/or push the individual below the poverty line. In addition, it is well known that major reliance on direct payments by the patient is likely to have a regressive impact on personal income distribution (Sanwald & Theurl, 2015; Wagstaff & Van Doorslaer, 2000). Finally, OOP payments act as a barrier for healthcare utilization and might have negative effects on long-term health status, especially for low-income individuals (Kiil & Houlberg, 2014). At the same time, if transaction costs of prepaid systems to safeguard insurance efficiency are substantial, cost sharing can improve social welfare. In addition, prepaid health care financing systems might cause substantial social losses through moral hazard ex ante and ex post and different schemes of OOP payments (e.g. deductibles, proportional cost sharing) may be used as an instrument to reduce these losses.

It is therefore not surprising that the role of prepayment is of high relevance in health care reform initiatives in many countries, especially within the broader concept of universal health care coverage initiated by the WHO (WHO, 2017). But effective strategies to implement and disseminate prepayment have to be based on systematic empirical knowledge about the determinants of health care financing.

In this paper we study economic, socio-demographic, political and institutional covariates of prepaid modes of health care financing in a worldwide country perspective. We refer to a panel data set comprising 154 countries covering the time period 2000–2015. We use three different indicators to capture the prepaid modes of health care financing: (i) the share of prepaid financing as percent of total current health care expenditure, (ii) the share of voluntary prepaid financing as percent of total prepaid financing, and (iii) the share of compulsory health insurance as percent of total compulsory prepaid financing. Aside using a static model we allow for a dynamic adjustment of the dependent variable and estimate a dynamic panel model including country and time fixed effects and controlling for endogeneity and autocorrelation of some regressors. As there exists evidence that prepaid health care financing might be closely related to the level of socio-economic development (Dieleman et al. 2017; Fan & Savedoff, 2014) we separately analyze prepaid health care financing for subsamples of rich and poor countries.

The paper contributes in several ways to the recent literature on the determinants of health care financing.^1^ First, instead of focusing on monetary values of health care financing (Ke et al. 2011) or OOP shares (Fan & Savedoff, 2014) this paper aims at providing deeper insights into financing structures rather than financing levels. Second, our analysis complements the available case studies on prepaid health care financing for selected countries (Ataguba & McIntyre, 2018; Barasa et al. 2017; Lagomarsino et al. 2012; McIntyre et al. 2018) by providing a global perspective on financing structures. Third, our analysis is based on most recent data on health care financing following the Global Health Expenditure Database of the WHO which is based on the System of Health Accounts 2011. The database covers the years 2000–2015 and provides information on health care financing from 189 countries. Fourth, the set of explanatory variables in this study comprises economic, socio-demographic, political and institutional factors and thereby extends the set of variables used in previous analysis substantially. Methodologically, as we use relative measures for describing the role and structure of health care financing we calculate the log-odds-ratios for the dependent variables before applying a standard fixed effects model and a dynamic fixed effects model.

Our results show that the structure of health care financing in previous years significantly determines its current structure. In addition, we find that the share of prepaid financing significantly increases while the share of voluntary prepaid financing significantly decreases with increasing governmental revenues and urbanization. While GDP/capita and regulatory quality significantly reduce the share of voluntary prepaid financing, development assistance for health shows a significantly positive impact on the share of voluntary prepaid financing. The share of compulsory health insurance increases with increasing GDP/capita and agricultural value added. The countries’ share of the elderly, the development of financial markets, the role of democracy in the past, and the countries’ overall education level are of minor importance for explaining the role and the structure of prepaid health care financing. The importance of the independent variables for explaining prepaid health care financing varies across subsamples of rich and poor countries.

The remainder of the paper is organized as follows. In Sect. 2, we describe the data sources as well as the dependent and explanatory variables. We also present some stylized facts which provide first insights into the structure, importance and temporal development of prepaid health care financing. Section. 3 outlines our estimation strategy and Sect. 4 presents results and a discussion. Finally, Sect. 5 concludes.

## Variables and data

### Dependent variables

We start with a short description of our data source and the definition of the indicators of prepaid financing used as dependent variables in our estimations. Our exclusive data source for the role and structure of prepaid health care financing is the Global Health Expenditure Database (GHED) of the WHO. The framework of this data base uses the System of Health Accounts (SHA) 2011. Data in the SHA 2011 specification are only available since the year 2000, so we take 2000 – 2015 as our sample period. The GHED provides two types of indicators, which inform on prepaid health care financing: indicators of sources of funds and indicators that describe financing arrangements. We use the second classification with minor modifications.

To picture the role and the structure of prepaid health care financing we separate four types of health care financing, namely (i) health care financed by the state government, (ii) health care financed by compulsory health insurance (this includes primarily social health insurance but also compulsory (private) health insurance schemes existing in some countries such as Switzerland), (iii) health care financed by voluntary prepaid schemes (this includes traditional private health insurance but also voluntary prepaid schemes offered by non-profit institutions and enterprise based prepaid financing schemes) and (iv) health care financed OOP. From an empirical point of view within the types (ii) and (iii) social health insurance respectively private health insurance are the dominant financing sources.

To examine the role and structure of prepaid health care financing we define three dependent variables:

1. Total prepaid financing as percent of total current health expenditures (*pre_che*),
2. Voluntary prepaid financing as percent of total prepaid financing (*vol_pre)* and
3. Compulsory health insurance as percent of compulsory prepaid financing (*hi_com*).

#### Total prepaid financing as percent of current health expenditures (pre_che)

The first indicator *pre_che* measures the relative importance of total prepaid health care financing compared to OOP payments. The choice of this variable is motivated by the important consequences of the trade-off between direct payments and prepaid financing for the organization of the health care financing process, for social welfare, equity and efficiency of health care financing and health care utilization. We use total current health expenditures as denominator. Total current health expenditure refers to all health goods and services used during a year to produce health care. The more volatile and future oriented category gross capital formation in the health care sector (investments in building & machinery & IT) is excluded. The numerator, total prepaid financing, refers to the GHED’s “financing arrangements” and includes: (i) prepaid arrangements financed by the government, (ii) prepaid arrangements financed by compulsory health insurance, (iii) prepaid arrangements by voluntary prepaid schemes.

#### Voluntary prepaid financing as percent of total prepaid financing (vol_pre)

The three prepaid financing mechanisms have in common that they separate the utilization of health care services from its financing and therefore include risk pooling effects. However, character and size of the pooling effects differ widely between and also within the three financing modes. A risk pool might be unitary, fragmented or integrated. Government financed risk pools might differ by their size, by the sort of taxes used for financing, by the role of external financing through intergovernmental fiscal relations, by the entitlements granted to the risk pool members, a.s.o. We capture one important facet of this heterogeneity of prepaid financing by our second dependent variable (*vol_pre*): It relates voluntary and compulsory prepaid financing. The two forms of prepaid financing differ widely in their scope of risk pooling. Risk pools financed by private health insurance are not homogenous. They differ in their reliance on experience rating and consequently in their character and amount of intertemporal and interpersonal risk pooling. But basically voluntary prepaid financing is based on the equivalence of health expenditure risk and individual contribution and therefore includes only a narrow form of risk pooling. Compulsory forms of prepaid financing cut this equivalence. They allow an extension of the intertemporal and interpersonal risk pooling and also include elements of income redistribution (Sinn, 1995).

In addition, risk pools based on compulsory financing face different advantages and challenges (e.g. transaction costs, problem of adverse selection and cream skimming, possible forms of pooling) compared to risk pools based on voluntary financing.

In the variable *vol_pre,* voluntary prepaid financing forms the numerator of the dependent variable. The denominator consists of total prepaid financing. In this context we prefer the separation by the criteria voluntary vs. compulsory over the criteria private vs. public, because the former is more important for the organization and effects of risk pooling.

#### Compulsory health insurance as percent of compulsory prepaid financing (hi_com)

The third indicator (*hi_com*) depicts the structure of compulsory prepaid financing. It relates compulsory health insurance and government based health care financing. The empirical literature reveals substantial differences between government- and social health insurance-financed health care systems (Wagstaff, 2010). These systems differ in the criteria of eligibility in the risk pool, in the financing instruments, in the administrative costs of health care financing, in the relationship to the health care providers and in the integration in the political system, with important consequences to efficiency and equity in health care financing and utilization and for the whole economy (e.g. labor market).

### Stylized facts

To motivate our empirical approach we start with a few stylized facts on worldwide health care financing with special focus on prepaid health care financing.

On a worldwide unweighted cross-country average, 40% of the current total health expenditures are government financed, 15% are financed by compulsory health insurance, 10% are financed by voluntary prepaid schemes (adding up to 65% of total prepaid financing) and 35% are OOP. This summarizes to a compulsory financing of 55% and a voluntary financing of 45%.

**Fig. 1:**
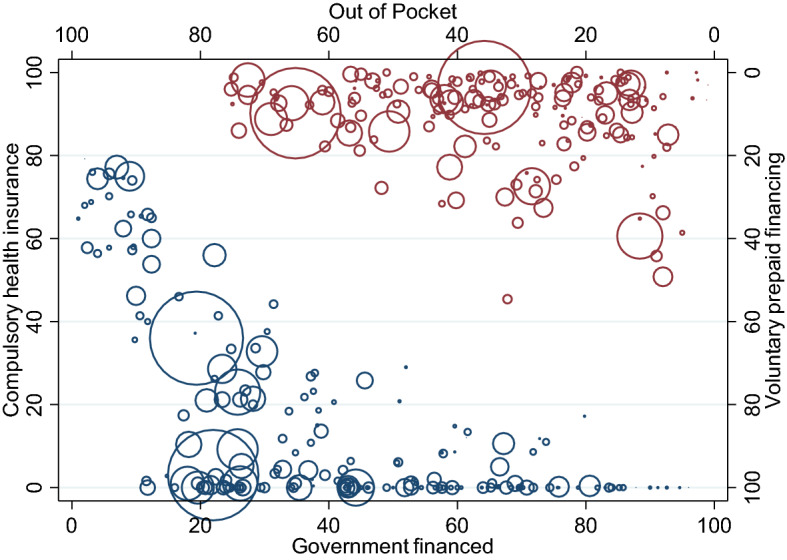
Types of health care financing as % of total current health care expenditures (years 2011–2015). The size of the circles pictures the countries’ population

Figure 1 pictures the four different sources of health care financing (excluding external help) in a worldwide perspective. All indicators are measured as percent of total current expenditures on health and are averaged over the five most recent years (2011–2015). Government financing and compulsory health insurance refer to compulsory forms of financing (starting in the bottom left corner of Fig. 1) while voluntary prepaid financing and OOP expenditures inform about the role of voluntary financing (starting in the top right corner of Fig. 1). The size of the circles refers to the countries' population.

Figure 1 reveals that there is a huge heterogeneity in the financing structure across countries. Compulsory health insurance of the three largest states, China, India and the USA, amounts to 36.0%, 3.0%, and 23.0%, respectively. Their government-financed health care account for 19.4%, 22.0% and 25.8%. With respect to the voluntary financed health care, the two Asian countries show low proportions of voluntary prepaid schemes (China: 3.6%, India: 9.8%) while in the USA 39.4% of current health expenditures are covered by such—in the case of the US mainly firm based insurance schemes. Reversely, OOP expenditures are in the USA with 11.6% relatively low compared to China (35.8%) and India (65.2%).

Figure 1 further highlights that although compulsory health insurance is zero or close to zero in several countries, the overall share of expenditures covered by public funds is at least around 12%. In particular, in 115 out of 189 countries either the share of compulsory health insurance or the share of government-financed health services is below 5% but even in these countries the overall share of compulsory financed health care ranges between 11.8% (Afghanistan) and 96.0% (Tuvalu). With respect to the voluntary prepaid schemes the figure shows that voluntary prepaid financing ranges between 0 and 55%. For 67 out of 189 countries, expenditures covered by voluntary prepaid schemes are below 5%. In contrast, the variation in the OOP payments is more pronounced. Finally, Figure 1 also pictures that the variation in OOP payments arises from the heterogeneity in publicly financed health care rather than from variations in private prepaid schemes.

To offer first insights into the relevance of the socio-economic development for prepaid health care financing we separate the sample into four country groups according to the World Bank’s classification of countries: low, lower middle, higher middle and high income countries. Comparing the shares for government financed health care as % of current total health expenditures across two income groups, rich (high and upper middle income countries) and poor (lower middle and low income countries) we find a balanced share, namely 40%, in both income groups. In contrast, the share of compulsory health insurance in rich countries is considerably higher than in poor countries (26% vs. 8%). The difference in the share of voluntary prepaid schemes between the two country groups is moderate: About 9% of the health care in rich countries is financed through voluntary prepaid schemes; for poor countries the share of voluntary prepaid financing amounts to 11%. The share of OOP payments is considerably higher in the low income group compared to rich countries (41% vs. 25%).

**Fig. 2:**
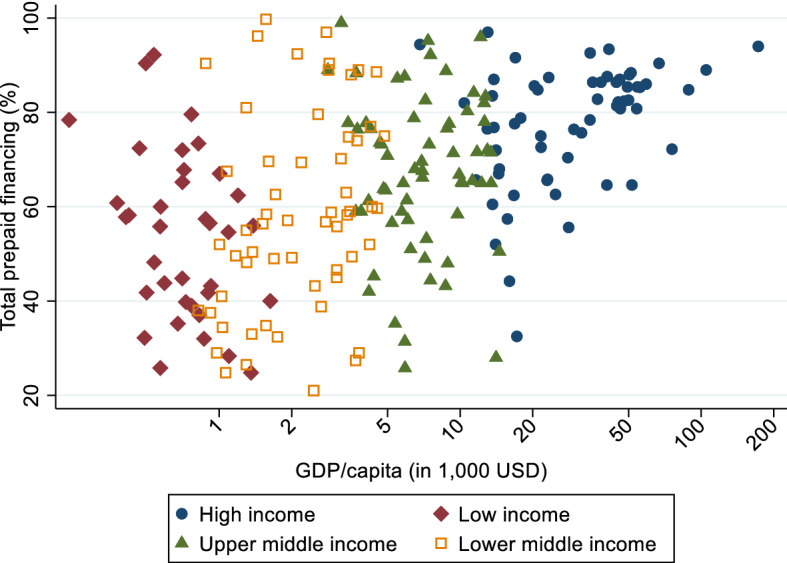
Total prepaid financing (% of current health expenditure) and GDP/capita; years 2011–2015. The label of the x-axis shows the GDP/capita in constant 2005 USD. The individual points in the graph show the logarithmic GDP/capita values

Figure 2 shows the relationship between total prepaid health care financing as % of current health expenditure and GDP per capita (averages over the time period 2011–2015). It reveals a huge heterogeneity in the prepaid health care financing, especially for low and lower middle income countries. Looking at the raw data we do not observe a strong effect of GDP on the role of prepaid health care financing. Only for high income countries there seems to be a positive effect of GDP. We examine this issue in detail in our empirical analysis by providing separate regressions for rich (high and upper middle income) and poor (lower middle and low income) countries.

**Fig. 3:**
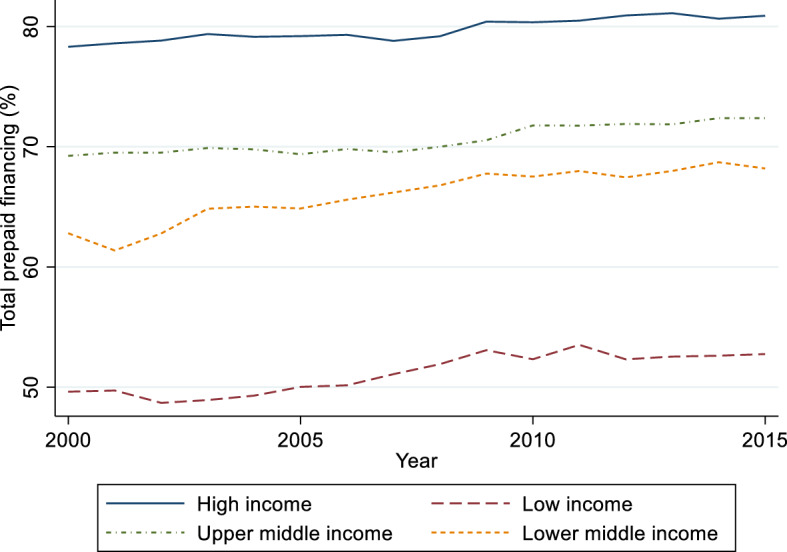
Total prepaid financing (% of current health expenditure), years 2000–2015. Income groups based on World Bank’s country classification in year 2000

Figure 3 pictures the development of total prepaid health care financing as % of current health expenditures in the time period 2000 – 2015 for the four income groups. Overall the role of prepaid financing rises in all income groups, but not monotonously. In the earlier years (2000–2010) the increase is higher in all groups of countries than in the years 2011–2015. In lower middle income countries the growth in the share of total prepaid financing is higher than in the other country groups. Convergence in the share of prepaid financing between the country groups during the time period seems to be only very modest.

**Fig. 4:**
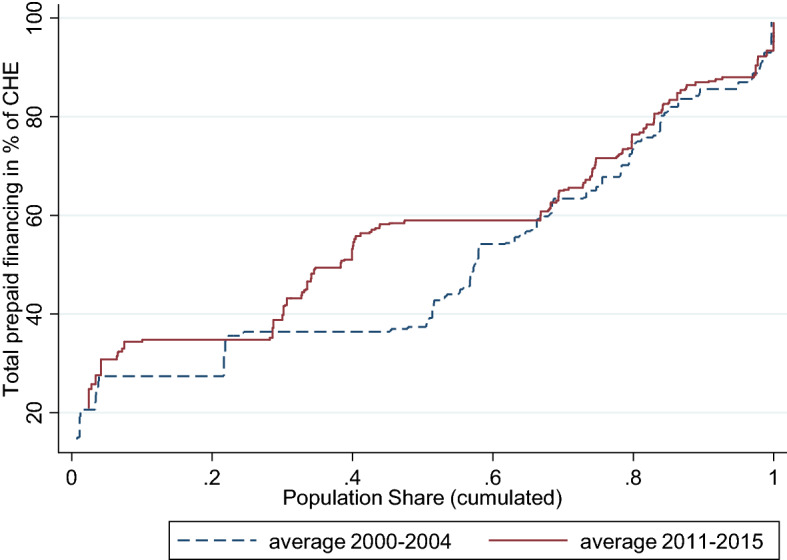
Worldwide prepaid health care financing; population weighted development over time. Countries are ordered on the x-axis by their share of total prepaid financing in ascending order

Figure 4 shows the worldwide development of total prepaid health care financing (y-axis) over time taking into account the countries’ population share (x-axis). The remaining source of health care financing, OOP payments, is defined as the difference between 100% and the share of the total prepaid plans. Countries are ordered on the x-axis by their share of total prepaid financing in ascending order. We plot the average share of prepaid financing in the starting period of our sample including data from 2000 – 2004 and in the last period including data from 2011 – 2015. The size of the countries can be derived from the horizontal length of the lines. Rather than focusing on the states but considering their population shares, this form of presentation allows population weighted statements on the role of total prepaid financing. In the most recent period, the median individual (from China) faces a share of total prepaid financing of 59%. For 62% of the world population the share of prepaid schemes is 50% or higher. Comparing the two time periods we can conclude that the majority of countries experience an increase in the share of prepaid plans and, consequently, a decrease in OOP payments over time. From a worldwide—population oriented—perspective the increase in China and India are especially remarkable (increase of prepaid share from 27.4% to 34.8% in India and 36.4% to 59% in China). A closer look at the changes also reveals a development of convergence of prepaid financing at a higher level.

### Explanatory variables

Theoretical insights and previous literature motivates the inclusion of various independent variables to explain the variation in the structure of health care financing. In the following, we discuss the expected influence of each explanatory variable on the three dependent variables *pre_che, vol_pre, hi_com* and present their data sources in Table 4 in the Appendix.

**Table 1:** Fixed effects estimation for total prepaid financing, voluntary prepaid financing and compulsory health insurance

	pre_che		vol_pre		hi_com	
	Static	Dynamic	Static	Dynamic	Static	Dynamic
Variables	(1)	(2)	(3)	(4)	(5)	(6)
L.pre_che_lo		0.844***				
		(0.033)				
L.vol_pre_lo				0.822***		
				(0.045)		
L.hi_com_lo						0.683***
						(0.077)
L.lngdppc_q1	−0.203	−0.034	−0.150	−0.144**	0.959***	0.144
	(0.140)	(0.044)	(0.276)	(0.066)	(0.337)	(0.150)
L.lngdppc_q2	−0.210	−0.034	−0.178	−0.141**	0.948***	0.134
	(0.137)	(0.043)	(0.273)	(0.066)	(0.331)	(0.149)
L.lngdppc_q3	−0.204	−0.033	−0.200	−0.144**	0.971***	0.141
	(0.135)	(0.042)	(0.269)	(0.065)	(0.327)	(0.149)
L.lngdppc_q4	−0.208	−0.030	−0.223	−0.151**	0.937***	0.130
	(0.133)	(0.041)	(0.265)	(0.064)	(0.320)	(0.150)
L.govrev	0.017***	0.002**	−0.015***	−0.006***	−0.003	−0.002
	(0.004)	(0.001)	(0.005)	(0.002)	(0.006)	(0.003)
L.lpr	−0.004	−0.000	−0.004	−0.004	−0.025	−0.006
	(0.008)	(0.002)	(0.011)	(0.004)	(0.020)	(0.007)
va_agri	0.008	0.000	0.014	0.004	0.030**	0.013**
	(0.006)	(0.002)	(0.010)	(0.002)	(0.014)	(0.006)
dah_cap	0.000	−0.001	0.006***	0.001*	−0.002	−0.003
	(0.003)	(0.001)	(0.002)	(0.001)	(0.006)	(0.003)
findev	−0.284	−0.029	0.217	0.256	−0.605	0.200
	(0.365)	(0.107)	(0.560)	(0.186)	(0.760)	(0.336)
popurb	0.021*	0.004	−0.034**	−0.002	0.013	0.006
	(0.011)	(0.003)	(0.017)	(0.005)	(0.025)	(0.009)
pop65	−0.037*	−0.007	−0.061	−0.001	−0.018	−0.027
	(0.020)	(0.006)	(0.045)	(0.011)	(0.057)	(0.022)
myschool	0.009	0.017*	0.018	−0.015	0.069	0.007
	(0.041)	(0.010)	(0.063)	(0.015)	(0.076)	(0.023)
democperm	0.010	0.069	−0.022	−0.038	0.234	−0.000
	(0.144)	(0.067)	(0.186)	(0.040)	(0.206)	(0.079)
regqual	0.049	0.026	−0.266**	−0.064*	−0.246*	−0.006
	(0.078)	(0.033)	(0.117)	(0.038)	(0.148)	(0.059)
Observations	2,303	2,135	2,226	2,043	1,390	1,185

Constant, time and country fixed effects not reported. Robust standard errors in parentheses. *** p < 0.01, ** p < 0.05, * p < 0.1. Dependent variable in columns (1) and (2): total prepaid financing as percent of total current health expenditures; dependent variable in columns (3) and (4): voluntary prepaid financing as percent of total prepaid financing; dependent variable in columns (5) and (6): compulsory health insurance as percent of compulsory prepaid financing

#### GDP/capita

Information on the constant (2005 USD) GDP per capita (*gdppc_cons*) are taken from the World Bank's World Development Indicators (WDI). We include the one year lag of GDP/capita in logs as explanatory variable as the contemporaneous variable might be endogenous with our dependent variables. The influence of the GDP/capita on *pre_che* depends on how risk preferences vary with changes in GDP/capita. As Cebula (2006), we assume that health insurance is a normal good. Hence, we expect an increase in the health insurance level with rising income. However, how increasing income affects the relative importance of prepaid financing in total current health expenditure and, hence, the share of prepaid financing is ambiguous. With respect to *vol_pre* we expect a positive impact of GDP/capita on voluntary prepaid financing. This is because preferences for product diversification, i.e., a variation in insurance packages, increase with income.

With respect to the *hi_com* there exist arguments for a positive as well as a negative impact of GDP/capita on the share of compulsory health insurance relative to compulsory prepaid financing. A negative relation between GDP/capita and *hi_com* is expected as tax-financed health care systems are characterized by a progressive tariff structure: If income rises, revenues rise disproportionately. By contrast, social health insurance systems generally have a proportional or regressive tariff structure. I.e., if income rises, revenues rise proportionally at most. Following this argumentation, it is expected that income will have a negative impact on the share of compulsory health insurance whereas the importance of tax-financed health systems increases. A positive influence of GDP/capita, on the other hand, is supported by the fact that insurance-based systems try to reflect the individual (group) preferences better than tax-financed systems, even when insurance-based systems are subject to compulsory insurance. Accordingly, the share of insurance-based systems should increase with increasing income.

#### Government revenue

Data on the general government revenue (*govrev*) is provided by the IMF, World Economic Outlook Database. We include the one year lag of government revenues (measured as % of GDP) as explanatory variable in order to reduce the potential endogeneity of *govrev*. Government revenue is seen as a proxy of the states’ financing potential and tax efforts (Gupta, 2007). If the public financing potential is high, the possibility of (public) prepaid financing increases. Therefore we expect a positive influence of governmental revenues on *pre_che* and a negative impact on *vol_pre*. Since government revenues comprise taxes and contributions to social health insurance the effect of this variable on *hi_com* is ambiguous.

#### Labor participation rate

Data on the labor force participation rate (*lpr*) is available from the World Bank's WDI. Similar to the GDP/capita, we include for endogeneity reasons the one year lag of the labor participation rate. The labor participation rate is expected to positively influence *pre_che* for several reasons. (i) An important precondition for prepaid financing is formal monetized income which arises from the individuals’ participation in the official labor market. (ii) The organization of prepaid financing systems often anchors at the existence of a workplace. Historically, this is particularly true for the traditional social health insurance systems in Europe. (iii) The higher the labor participation rate, the lower the shadow economy and the higher the coverage provided by prepaid financing systems. Each of these three statements applies to total prepaid financing in general but particularly holds for compulsory prepaid systems so that the influence of the labor participation rate on *vol_pre* is expected to be negative. The positive influence of the labor participation rate on *hi_com* can be justified as follows: The financing of compulsory health insurance systems highly depends on the performance of the labor market (i.e., wage levels, number of employees). Taxation on the other hand uses a broader contribution base and includes not only labor income but also capital income, consumption and wealth. Therefore, an increase in the labor force participation rate should lead to an increase in the share of compulsory health insurance.

#### Agricultural value added

For the variable agricultural value added, which we took from the World Bank’s WDI, we expect a negative impact on *pre_che*. This can be explained by a characteristic of the agricultural sector, namely that production and consumption are not spatially separated (family-run farms). This firm structure enables informal forms of health risk coverage via family members (Dercon, 2002; Gertler & Gruber, 2002). The higher the proportion of informal coverage the lower is the demand for formal prepaid coverage. Furthermore, workers in agricultural firms receive a substantial part of their income as payment in kind (i.e., in form of natural produce). Payments in kind only have implicit prices and are therefore a poor contribution base, especially for compulsory prepaid systems. The above arguments also apply for *vol_pre* (i.e. the comparison of voluntary prepaid vs. compulsory prepaid) which let expect a positive influence of the agricultural sectors’ size on *vol_pre*. Finally, the expected influence of agricultural value added on *hi_com* is ambiguous.

#### Development assistance for health (DAH) per capita

Information on development assistance for health, measured in constant 2014 USD, is provided by the Institute for Health Metrics and Evaluation (IHME, 2017). We divide this variable by the countries’ population (taken from the WDI) to achieve the development assistance for health per capita (*dah_cap*). DAH will, with a few exceptions, flow into the prepaid sector and will be used to finance public projects. DAH is therefore expected to have a positive influence on *pre_che* and a negative impact on *vol_pre*. With regard to *hi_com*, the expected sign of DAH is negative. States are interested in using DAH to increase their state budget and thus gain political benefits. I.e., DAH will flow into health projects initiated by the government rather than initiated by compulsory health insurance. One exception is projects, especially in countries with precarious political conditions, which are aimed at strengthening citizens' rights (strengthening social rights; contractual security, social security). In such countries systems autonomous from the state are more likely to strengthen civil rights and to receive international financial support.

#### Financial development index

There exists theoretical and empirical evidence that the insurance sector is strongly influenced by the financial sector (Feyen, Lester & Rocha, 2011). We therefore include the financial development index in our estimation. Information on financial development (*findev*) is taken from an IMF Working Paper by Svirydzenka (2016). In contrast to the previous literature which approximates financial development by indicators on financial depth only—such as the ratio of private credit to GDP – Svirydzenka (2016) uses nine indices which picture how developed financial institutions and financial markets are in terms of their depth, access and efficiency. These indices are then aggregated into an overall index of financial development. We argue that the level of financial development influences the role und structure of prepaid health care financing. We specifically expect that voluntary prepaid financing is higher in countries with well-developed financial institutions/markets. A well-developed financial sector reduces the transaction costs of prepaid health insurance and allows the building of capital stocks (funded systems). In addition they provide additional instruments for risk allocation for the providers of prepaid plans. We do not expect a substantial effect of a country’s financial development on the structure of compulsory prepaid health care financing (social health insurance vs. taxes). These systems rely on pay-as-you-go financing and the need of funded financing is of little importance.

#### Urban population

Data on the urban population (*popurb*) are taken from the World Bank’s WDI series. A high proportion of the population in urban areas should influence *pre_che* and *vol_pre* positively. This can be explained by lower transaction costs and the presence of peer effects in agglomerations so that the development and organization of compulsory and voluntary prepaid systems in urban areas works better compared to rural regions. The same argument let expect a positive influence of urbanization on *hi_com*: Transaction costs are higher for compulsory health insurance than tax financed health coverage so that compulsory health insurance benefits more from urbanization than tax financed systems.

#### *Share of population aged 65* + 

The share of population older than 64 years (*pop65* +) is again taken from the World Bank’s WDI. Two arguments can be brought forward which let assume a positive influence of the population share of the 65 + on *pre_che*. (i) As the proportion of people over 64 years of age increases, there is political pressure to expand prepaid systems (especially compulsory prepaid systems), because the elderly benefit from redistribution within prepaid systems. (ii) The savings of older people are built up via prepaid systems. The larger the proportion of the population over 64, the higher is on average explicit (houses, private insurance) and implicit (insurance claims) wealth. From argument (i) it further follows, that a large share of elderly will negatively influence *vol_pre*. The influence of the elderly on *hi_com* is ambiguous.

#### Education

Education is captured via the average years of schooling (*myschool*) reported in the UNs Human Development Reports. Education, measured as mean years of schooling, is expected to positively influence *pre_che* for the following reasons. (i) Education increases individual risk aversion so that educated people have a higher demand for protection. (ii) Educated are more patient and are willing to delay gratification; their rate of time discounting is lower which results in a higher demand for future coverage. (iii) Education increases understanding of the functioning and usefulness of prepaid systems. Argument (iii) also justifies a positive influence of education on *vol_pre*. Since the cognitive challenges in voluntary prepaid schemes are higher compared to compulsory financing systems, voluntary prepaid systems have significantly higher transaction costs than compulsory prepaid systems. Education lowers these transaction costs. Information costs are also larger in social health insurance systems than in tax-financed systems so that education is expected to also positively influence the share of compulsory health insurance over tax-financed coverage, *hi_com*.

#### Percentage of democratic years (1980–2015)

An indicator (*polity2*) of the countries policy regime is taken from the Polity IV project, Political Regime Characteristics and Transitions, 1800–2013. We use this information to calculate the fraction of democratic years (*democperm*) since 1980 up to year *t* according to Besley and Kudamatsu (2006).

(Public) prepaid systems involve redistribution (risk redistribution, income redistribution) the majority of the population benefits from. In autocratic systems, maintaining political power has priority. Political power is achieved by rewarding system-relevant target groups (civil servants, the military, the rich, etc.) so that these groups have a low interest in a general redistribution (Besley & Kudamatsu, 2006). In democratic systems, on the other hand, the opinion of the median voter is decisive. *Pre_che* is therefore expected to be the higher the more years (measured from 1980–2015) a state has a democratic government. Olsen (1982) provides another argument for a positive influence of democratic systems on prepaid systems: The expansion and development of prepaid systems takes time. The longer a democratic government is in office, the more pronounced prepaid financing systems will be. The argumentation for a negative influence of democracies on *vol_pre* (and therefore in favor of compulsory prepaid) is similar to that for *pre_che*. In addition, voluntary prepaid systems include only protection against cost of illness but no redistribution of income, whereas compulsory systems that were financed on an income-oriented basis also offer protection against income fluctuations (= redistribution of income). Hence, the median voter will opt for compulsory prepaid financing.

Two arguments suggest a negative influence of the proportion of years a democratic government is in office on *hi_com*. (i) In autocratic systems, compulsory health insurance is an instrument of the ruling group to make systematically important population strata (civil servants, military) immune against social revolutions (Besley & Kudamatsu, 2006). (ii) Social security systems are particularistic and characterized by regressive or proportional tariffs, whereas tax-financed systems are unitary and tariffs are mostly progressive. The median voter would prefer c.p. tax-financed systems with more egalitarian coverage.

##### Regulatory quality

An index (*regqual*) reflecting the countries' regulatory quality is provided by the World Bank’s Worldwide Governance Indicators (WGI) Project. According to the WGI’s definition regulatory quality *“captures perceptions of the ability of the government to formulate and implement sound policies and regulations that permit and promote private sector development.”* Hence, regulatory quality refers to the perceived efficiency of the government which influences para-fiscal institutions as well as private companies through legislation. Regulatory quality is expected to positively influence prepaid systems. Such systems are future-oriented and binding. They therefore require trust in contractual stability. High quality governmental regulations are an essential basis for this trust. The higher the quality of regulation, the higher the confidence in and demand for prepaid financing schemes and, following, the higher will be *pre_che*. For *vol_pre* and *hi_com* the effect of regulatory quality is ambiguous.

## Estimation strategy

### Empirical model and estimation

Since the three dependent variables *pre_che, vol_pre* and *hi_com* are shares, we first calculate the log-odds-ratio for the dependent variables and apply a standard fixed effects model on the linearized dependent variables.^2^ Our main specification reads as

1 $$ln\left(\frac{{y}_{it}}{1-{y}_{it}}\right)={\beta }_{0}+{{\varvec{\beta}}}_{1} {{\varvec{e}}{\varvec{c}}{\varvec{o}}{\varvec{n}}}_{it}+ {{\varvec{\beta}}}_{2}\boldsymbol{ }{{\varvec{d}}{\varvec{e}}{\varvec{m}}{\varvec{o}}}_{it}+ {{\varvec{\beta}}}_{3} {{\varvec{p}}{\varvec{o}}{\varvec{l}}}_{it}+{{\varvec{\delta}}}_{{\varvec{t}}}+{{\varvec{\theta}}}_{{\varvec{i}}}+ {\varepsilon }_{it}$$

with *y*_*it*_ either representing the share of total prepaid financing as percent of current health expenditures (*pre_che*), the share of voluntary prepaid financing as percent of total prepaid financing (*vol_pre*) or the share of compulsory health insurance as percent of compulsory prepaid financing (*hi_com*). *i* stands for country and *t* indicates the year of observation. The vector ***econ*** comprises the general government revenue as % of GDP (*govrev*), the labor force participation rate (*lpr*), the agricultural value added as percent of GDP (*va_agri*), the countries’ per capita development assistance for health (*dah_cap*) and an index capturing the countries’ financial development (*findev*). Finally, the vector ***econ*** also includes the log-transformed GDP per capita in constant 2005 US$. In order to allow for a non-linear influence of GDP/capita on the role and structure of prepaid health care financing, we include a separate regressor for each quartile of the logarithmic GDP/capita variable (*lngdppc_q1, lngdppc_q2, lngdppc_q3, lngdppc_q4*).

The vector ***demo*** includes the proportion of urban population *(popurb*), the proportion of people aged 65 or above (*pop65* +) and mean years of schooling (*myschool*). The vector ***pol*** collects information on the percentage of democratic years from 1980–2013 (*democperm*) and an index for regulatory quality (*regqual*). ${\delta }_{t}$ represents year dummies and controls for time specific influences that apply to all countries and the set of country dummies, ${\theta }_{i},$ captures influences which are fixed across countries. Finally, ${\varepsilon }_{it}$ represents the remainder error. To ensure exogeneity of specific explanatory variables with respect to the error term we include one-year lags of *lngdppc_q1- lngdppc_q4*, *govrev*, and *lpr.*

In addition to the static fixed effects model we estimate a dynamic fixed effects model developed by Hsiao et al. (2002). Kripfganz (2016) describes the Stata command xtdpdqml which allows for an easy implementation of this quasi-maximum likelihood (QML) estimation for linear panel data models. Compared to the system-GMM-estimator used in Ke et al. (2011) which instruments the endogenous lagged dependent variable by its lags, Kripfganz’s routine models initial observations of the dependent variable as a function of observed variables.

Including the lagged dependent variable, the specification for the linear panel data model is

2 $$ln\left(\frac{{y}_{it}}{1-{y}_{it}}\right)={\gamma }_{0}+{\lambda \left(\frac{{y}_{it-1}}{1-{y}_{it-1}}\right)}+{\gamma }_{1}{{\varvec{e}}{\varvec{c}}{\varvec{o}}{\varvec{n}}}_{it}+ {{\varvec{\gamma}}}_{2}\boldsymbol{ }{{\varvec{d}}{\varvec{e}}{\varvec{m}}{\varvec{o}}}_{it}+ {{\varvec{\gamma}}}_{3} {{\varvec{p}}{\varvec{o}}{\varvec{l}}}_{it}+{{\varvec{\eta}}}_{t}+{{\varvec{\phi}}}_{{\varvec{i}}}+ {\nu }_{it}$$

Using Kripfganz’s stata routine *xtdpdqml*, the actual estimation of Eq. (2) is performed on its first-differences which removes the time invariant parameters ${{\varvec{\phi}}}_{{\varvec{i}}}. {{\varvec{\eta}}}_{t}$ denotes time fixed effects and ${\nu }_{it}$ stands for the error term.

## Results

### Descriptive statistics

Our starting point is a panel data set with 3024 observations for 189 countries over 16 years (2000–2015). Of these 189 countries, 35 countries cannot be included in the analysis because information on at least one of the explanatory variables is missing in each year. This data limitation results in a sample of 154 countries. For 126 out of the 154 countries, information on dependent and explanatory variables is complete in each of the 16 years. For the remaining 28 countries, data for individual years is missing. However, as the minimum observation period even for these countries is 3 years, we keep them in our analysis. The final data set which the empirical analysis is based on includes 2,303 observations of 154 countries and 16 years (2000–2015).^3^ Table 5 in the Appendix summarizes the descriptive statistics of the dependent variables as well as explanatory variables used in the subsequent empirical analysis.

**Table 2:** Fixed effects estimation for total prepaid financing, voluntary prepaid financing and compulsory health insurance—rich countries

	pre_che		vol_pre		hi_com	
	Static	Dynamic	Static	Dynamic	Static	Dynamic
VARIABLES	(1)	(2)	(3)	(4)	(5)	(6)
L.pre_che_lo		0.809***				
		(0.058)				
L.vol_pre_lo				0.849***		
				(0.064)		
L.hi_com_lo						0.770***
						(0.049)
L.lngdppc_q3	0.025	0.102	−0.442	−0.039	0.894	0.112
	(0.176)	(0.067)	(0.405)	(0.112)	(0.561)	(0.215)
L.lngdppc_q4	0.010	0.103	−0.457	−0.044	0.852	0.106
	(0.172)	(0.067)	(0.399)	(0.111)	(0.551)	(0.214)
L.govrev	0.012*	0.004	−0.020**	−0.003	−0.002	−0.011**
	(0.006)	(0.003)	(0.009)	(0.003)	(0.018)	(0.005)
L.lpr	0.014	0.005	0.017	0.007	−0.016	−0.007
	(0.010)	(0.003)	(0.023)	(0.006)	(0.018)	(0.008)
va_agri	0.012	0.009	−0.061*	−0.021	0.065	0.031
	(0.024)	(0.010)	(0.035)	(0.013)	(0.050)	(0.026)
dah_cap	0.004***	0.001***	0.002***	0.001	0.005	0.011
	(0.000)	(0.000)	(0.001)	(0.001)	(0.012)	(0.015)
findev	−0.095	0.010	1.701**	0.539**	−1.495	0.064
	(0.401)	(0.124)	(0.668)	(0.244)	(1.022)	(0.327)
popurb	0.009	−0.003	−0.031	−0.007	−0.027	0.015**
	(0.012)	(0.004)	(0.022)	(0.004)	(0.033)	(0.008)
pop65	−0.100**	−0.024*	0.034	0.016	−0.121	−0.046**
	(0.039)	(0.013)	(0.051)	(0.013)	(0.074)	(0.020)
myschool	0.007	0.017*	0.009	−0.005	0.146	0.083**
	(0.036)	(0.010)	(0.063)	(0.019)	(0.118)	(0.041)
democperm	−0.057	0.077	0.055	−0.078	0.585**	0.272*
	(0.177)	(0.062)	(0.341)	(0.129)	(0.245)	(0.154)
regqual	−0.130	0.006	−0.136	0.011	−0.515**	−0.063
	(0.112)	(0.030)	(0.170)	(0.047)	(0.224)	(0.070)
Observations	853	796	826	771	574	505

Constant, time and country fixed effects not reported. Robust standard errors in parentheses. *** p < 0.01, ** p < 0.05, * p < 0.1. Dependent variable in columns (1) and (2): total prepaid financing as percent of total current health expenditures; dependent variable in columns (3) and (4): voluntary prepaid financing as percent of total prepaid financing; dependent variable in columns (5) and (6): compulsory health insurance as percent of compulsory prepaid financing

### Determinants of total prepaid financing as percent of current health expenditures

Columns (1) and (2) of Table 1 present the results for the role of prepaid financing, i.e., the share of total prepaid financing as percent of current health expenditures. The significantly positive coefficient of governmental revenues, which serves a proxy for the countries’ financing potential (Gupta, 2007) meets the expectations: The higher the scope for public financing the higher is the share of total prepaid financing. When we allow for a dynamic adjustment of the dependent variable and estimate a dynamic panel model governmental revenues still turn out as a significant determinant of prepaid health care financing. As expected, also the level of prepaid health care financing last year decisively influences today’s share. Of minor importance but in line with our expectation is the positive influence of urbanization and education on prepaid health care financing indicating that prepaid schemes are easier to implement in agglomerations and that educated people have higher demand for insurance coverage. In contrast, the weakly significant and negative influence of the share of the elderly contradicts the argument that the political pressure to expand the prepaid system increases with a growing share of the older population.

The influence of the remaining explanatory variables cannot be precisely estimated.

### Determinants of voluntary prepaid financing as percent of total prepaid financing

The analysis of the second dependent variable gives insights into the structure of total prepaid financing. The majority of prepaid financing is compulsory while voluntary prepaid financing plays a minor role.

As outlined above, governmental revenues picture the financial scope of governments to finance health care. Hence, we expect that in countries with higher governmental revenues compulsory prepaid financing is more important than voluntary financing. We find support for this hypothesis: Voluntary prepaid financing and, hence *vol_pre*, is the lower the higher governmental revenues are.

The significantly positive coefficient on development assistance for health indicates that this type of funds particularly supports voluntary health insurance systems. This positive impact does not meet our primary expectations, namely, that DAH will be mainly used to finance public projects. 

A possible explanation for this unexpected relationship can be derived from the data. In about 60% of the countries that received DAH, autocratic governments have been prevalent in the past 25 years. As already argued earlier, DAH in such countries is likely to aim at strengthening citizens’ rights by supporting voluntary, and likely non-governmental, financing arrangements.

The argument of easier access to and lower transaction costs for prepaid financing in urban regions should be particular valid for voluntary risk pools. Following, voluntary risk pooling should be easier in urban regions. The significantly negative impact of the urban population on voluntary prepaid financing in the static model does not meet this expectation.

A country’s regulatory quality does not only influence the public sector but also affects sectors which are under state supervision as well as private sectors via legislation. The significantly negative effect of regulatory quality on voluntary prepaid financing indicates that compulsory (public) financing is favored over voluntary financing the higher the regulatory quality is.

Referring to the estimates using the dynamic model, column (4) of Table 1 reveals that the GDP/capita negatively impacts the share of voluntary prepaid financing. Interestingly, the levels of the coefficients on the *GDP/capita* quartiles imply a nearly identical effect of GDP/capita across the different income ranges on voluntary prepaid financing. This significantly negative influence contradicts our expectation that preferences for product diversification and, hence, for voluntary prepaid financing, increases with increasing income. The lagged dependent variable again indicates that the share of voluntary prepaid financing today is crucially determined by its share in the previous year.

### Determinants of compulsory health insurance as percent of compulsory prepaid financing

The third dependent variable describes the trade-off between compulsory health insurance and tax-financed health care. The sample size is reduced to 1390 observations in the static model and 1185 in the dynamic model due to the fact that in some countries a compulsory health insurance does not exist.

The output of the static model presented in Column (5) of Table 1 shows that the share of compulsory health insurance increases with increasing GDP/capita. Again, the coefficients are of similar size across the different quartiles of the GDP/capita distribution. This positive effect indicates that wealthy nations prefer insurance-based systems which are superior in reflecting individual risk preferences compared to tax-financed systems. The negative impact of high regulatory quality on the share of compulsory health insurance may result from higher preferences for governmental health care financing over social health insurance once the regulatory quality of the public sector is high. Furthermore, the results in columns (5) and (6) of Table 1 suggest that the ratio of compulsory health insurance over taxed financed health care significantly increases with agricultural value added. In the dynamic model we again find that the lagged dependent variable positively impacts the share of compulsory health insurance.

### Prepaid health care financing in rich vs. poor countries

In this section we examine whether prepaid health care financing differs across rich and poor countries. For distinguishing between these two country groups we refer to the World Bank’s classification of countries. We assign high and upper middle income countries to the group of rich countries. Lower middle and low income countries form the group of poor countries.

The results for the two subsamples of rich (Table 2) and poor (Table 3) countries are largely in line with the results of the base regressions in Table 1. However, the importance of the individual determinants for prepaid health care financing varies across the subsamples.

**Table 3:** Fixed effects estimation for total prepaid financing, voluntary prepaid financing and compulsory health insurance—poor countries

	pre_che		vol_pre		hi_com	
	Static	Dynamic	Static	Dynamic	Static	Dynamic
Variables	(1)	(2)	(3)	(4)	(5)	(6)
L.pre_che_lo		0.832***				
		(0.033)				
L.vol_pre_lo				0.809***		
				(0.068)		
L.hi_com_lo						0.592***
						(0.104)
L.lngdppc_q1	−0.170	−0.076	−0.060	−0.187**	0.565	0.110
	(0.179)	(0.064)	(0.381)	(0.084)	(0.408)	(0.172)
L.lngdppc_q2	−0.174	−0.074	−0.094	−0.189**	0.563	0.105
	(0.175)	(0.063)	(0.378)	(0.086)	(0.401)	(0.172)
L.lngdppc_q3	−0.161	−0.069	−0.107	−0.192**	0.569	0.105
	(0.172)	(0.062)	(0.376)	(0.085)	(0.399)	(0.171)
L.govrev	0.019***	0.001	−0.015***	−0.005*	−0.002	0.002
	(0.004)	(0.001)	(0.006)	(0.003)	(0.006)	(0.003)
L.lpr	−0.019*	−0.005	−0.003	−0.004	−0.022	−0.005
	(0.010)	(0.003)	(0.013)	(0.005)	(0.025)	(0.007)
va_agri	0.008	−0.001	0.015	0.006**	0.019	0.010
	(0.006)	(0.002)	(0.010)	(0.003)	(0.014)	(0.007)
dah_cap	−0.004	−0.002	0.010***	0.002*	−0.002	−0.002
	(0.003)	(0.001)	(0.003)	(0.001)	(0.006)	(0.003)
findev	−0.677	−0.069	−1.055	−0.318	0.094	0.194
	(0.557)	(0.185)	(0.909)	(0.287)	(1.038)	(0.709)
popurb	0.031**	0.007**	−0.037*	−0.003	0.040	0.017
	(0.014)	(0.003)	(0.020)	(0.007)	(0.028)	(0.015)
pop65	0.002	−0.008	−0.129*	−0.018	0.145	0.056
	(0.040)	(0.011)	(0.072)	(0.020)	(0.122)	(0.052)
myschool	−0.045	−0.004	0.056	0.014	−0.008	−0.051
	(0.064)	(0.015)	(0.105)	(0.024)	(0.086)	(0.035)
democperm	0.090	0.078	−0.156	−0.149***	0.346	0.032
	(0.164)	(0.077)	(0.194)	(0.057)	(0.264)	(0.141)
regqual	0.153	0.033	−0.387***	−0.119***	−0.007	0.077
	(0.093)	(0.048)	(0.142)	(0.045)	(0.170)	(0.078)
Observations	1,436	1,326	1,386	1,261	812	676

Constant, time and country fixed effects not reported. Robust standard errors in parentheses. *** p < 0.01, ** p < 0.05, * p < 0.1. Dependent variable in columns (1) and (2): total prepaid financing as percent of total current health expenditures; dependent variable in columns (3) and (4): voluntary prepaid financing as percent of total prepaid financing; dependent variable in columns (5) and (6): compulsory health insurance as percent of compulsory prepaid financing

In particular, we find for both rich and poor countries that in the dynamic models the lagged dependent variables always reveal a significantly positive influence on the current structure of health care financing. Governmental revenue positively influences *pre_che* and negatively impacts *vol_pre.* For rich countries, governmental revenue also reveals a negative impact on *hi_com*. The positive impact of urbanization on *pre_che* and its negative impact on *vol_pre* which we observe in the base results seems to be driven by the poor countries in the sample: We find a significant impact of the share of urban population on *pre_che* and *vol_pre* for the poor subgroup only.

In the poor sample, regulatory quality is a decisive factor that negatively impacts *vol_pre* only while in rich countries solely *hi_com* significantly decreases with increasing regulatory quality. Development assistance for health influences *vol_pre* positively in both subsample. For rich countries development assistance for health also significantly increases *pre_che*. The positive sign for *pre_che* in rich countries is in line with our expectations. However, we expected a stronger effect for poor than rich countries, as DAH are primarily intended to support public health programs in developing countries.

With respect to the importance of the countries’ socio-economic development captured via the GDP/capita the results in Table 3 suggest that the significant negative impact of GDP/capita on *vol_pre* in the base regression is driven by the poor countries in the sample. Only for the subsample of poor countries we find a statistically significant and negative impact of GDP/capita on *vol_pre* whereby the size of the coefficients does not considerably vary across the GDP/capita quartiles. For rich countries the GDP/capita does not influence the structure of prepaid health care financing at all.

Our base regressions for the total sample (Table 1) as well as for the subsample of poor countries (Table 3) imply that a country’s financial development does not significantly determine the structure of prepaid financing. However, the results in Table 2 suggest a significantly increasing share of *vol_pre* with increasing financial development in rich countries. This positive relation is in line with our expectation that well-developed financial markets ease voluntary prepaid financing.

The share of the elderly seems to be primarily relevant for the prepaid health care financing structures in the rich countries, while this variable plays a minor role for the financing structures in the poor subsample: Table 2 shows that *pre_che* and *hi_com* decreases with an increasing share of people aged 65 or above. For poor countries this negative influence can only be observed for *vol_pre*.

The last comparison between the main regressions based on the total sample vs. the subsamples we would like to address is the role of a country’s policy regime (measured via the percentage of democratic years in the years 1980–2015). When we use the total sample the policy regime does not play a role at all for prepaid health care financing. However, once we split the sample and distinguish between rich and poor country groups we find a significantly positive impact of democratic governments on *hi_com* in rich countries and a significantly negative impact on *vol_pre* in poor countries.

As in the base model, the remaining explanatory variables only reveal a weak or no impact on the health care financing structures.

## Discussion

A comparison of the results from this study with the results from the more recent previous literature is only possible to a very limited extent. This is because we differ from this literature in several areas. First, our analysis focuses on structures in the financing of health care expenditures rather than on nominal values. Two of the dependent variables (namely *vol_pre* and *hi_com*) have not been analyzed before. Only the variable *pre_che* is comparable to one of the variables used in Fan and Savedoff (2014), namely to the OOP share of total health spending. However, aside the different time frame and geographical coverage, Fan and Savedoff (2014) use a smaller set of explanatory variables (incl. GDP per capita, governmental expenditures as % of GDP, population of 60 + , and combinations of country and time dummies) which makes a comparison of the results difficult. What we can say is that our results are in line with respect to the three included explanatory variables in Fan and Savedoff (2014), considering that our dependent variable is not the share of OOP expenditures over total health expenditure rather than the share of prepaid health care financing in current health expenditures (i.e., the OOP expenditures are included in the denominator).

Second, the current study uses a more comprehensive set of explanatory variables than previous literature to describe the countries’ institutional, socio-demographic and economic characteristics. Third, our study uses current data (2000–2015) from the WHO's Global Health Expenditure Database (GHED). One of the improvements of the current data in their system of health accounts is that it is now feasible to distinguish between current health expenditures and capital health expenditure.

Fourth, methodologically, we use log-odds-ratios of the dependent variables so that we can apply a standard fixed effects model and a dynamic fixed effects model. Our empirical outcome reveals a few significant determinants for the respective dependent variables which we believe is mainly due to our estimation strategy: (i) We focus on within country variation by including country and time fixed effects which control for all country and time specific factors. (ii) The dependent variables picture the structure of health care financing and are measured in relative values (shares). Common tendencies comprised in variables such as GDP per capita, population 65 + , or education for the numerator (e.g. nominal prepaid health care expenditures per capita) and the denominator (e. g. nominal total current health expenditures per capita) are therefore cancelled out. For example, from previous empirical research it is well known, that GDP per capita is a good predictor for the absolute level of health expenditure per capita and consequently for health care financing (Gerdtham & Jönsson, 2000; Ke et al. 2011). Our results indicate that this is not the case when we look at the relationship of GPD per capita and the share of total prepaid health care financing.

Fifth, the data to answer our research question is at the country level. These macro data represent the aggregates of individual decisions and make it difficult to adequately map the relevant constraints of the individual decision process. Although we use a quite comprehensive set of explanatory variables important determinants of the structure of health care financing are not included in our analysis due to the non-availability of valid data. This particularly holds for two variables: information on income (wealth) inequality and information on the distribution of health expenditure risks within the population. We assume that the choice between the different financing options is based on an individual economic trade-off between the expected benefits of a financing option (which are closely related to the individual health risks) and the financing burden (which is strongly related with income). Reliable information on income inequality (measured by the Gini-coefficient) is totally missing for many countries, resilient time series information over the whole time period 2000–2015 is missing for almost all countries. Information on health risks distribution is even worse so that the necessary matching of the income and health risk distribution is not possible. In addition, we expect that the individual decision on the different financing options is not based on present/transitory individual characteristics only but includes at least elements of a life cycle perspective, a dimension which is not captured by the used macro data either. Individual data, or at least macro indicators that reflect the distribution of characteristics within the population of a country, would allow a more detailed analysis.

An assessment of the empirical results also needs a discussion of the definition of the dependent variables. Overall, the indicators *pre_che, vol_pre* and *hi_com* offer (highly) aggregated information on the role and structure of prepaid health care financing. The use of financing shares on a macro basis measured by realized monetary values as an indicator for the role of prepaid financing has important implications for the interpretation of the data. The well-known WHO-cube of health care financing separates three dimensions of coverage by prepaid schemes: (i) the proportion of the population, which has coverage (breadth of coverage), (ii) the proportion of the services covered (depth of coverage), and (iii) the proportion of the costs covered (height of coverage). Our data set does not allow empirical statements regarding the role of the three dimensions of coverage by prepaid plans. The same share of prepaid financing in two countries is compatible with very different combinations of the three dimensions. In addition, these combinations will differ within the three modes of prepaid financing. This is an important limitation for an interpretation of the empirical picture from a normative perspective. In this respect it is also important to emphasize that we cannot draw conclusions whether the health care services–the benefit catalogue—financed by prepaid plans are appropriate, effective, efficient and further the goal of equity or not.

The different dimensions of performance are important from the perspective of universal health coverage. This concept does not only include the financial dimension but also the availability of an appropriate basket of health care services. Empirical work by Wagstaff et al. (2015) for 24 developing countries offers strategies to enlarge our perspective. They present a workable definition of the universal health concept and formalize an index, which includes two dimension: (i) financial protection (nobody should suffer financial hardship as a result of needing care) and (ii) service coverage (everyone, irrespective of his ability to pay should have access to the needed services). It is obvious, that the role of specific forms of prepaid financing as we focus on is a good proxy for the financial domains. We admit, that the enlargement of our concept of financial protection by the dimension “service coverage” is necessary, but due to the lack of worldwide data on service coverage we have to leave the implementation of this step to future work.

Preliminary results with an approach similar to that of Wagstaff et al. (2015) offers a paper by Feigl and Ding (2013). The authors study economic, social and political determinants of universal health coverage in a longitudinal study of 194 countries and also use a formal indicator of coverage including the following domains: (i) percentage of the population included in a public or private prepaid health care plan, (ii) access to health care services measured by the percentage of skilled attendance at birth, (iii) legal universal coverage. Legal universal coverage was identified by studying country specific legislative texts which indicated whether the entire population/citizenry was covered in the health plan and was granted access to a core set of services. The description of the included domains reveals the fundamental difference of the Feigl and Ding (2013) study and our study thereby leaving aside differences in the econometric approach: We study realized monetary values for the role of prepaid financing while Feigl & Ding focus at the existence of entitlements.

Finally, our data does not reveal information on the relationship between the different prepaid financing options and on the relationship between prepaid financing and OOP. Basically the different forms can be substitutes or complements. This is particularly true for social health insurance and voluntary prepaid financing. For the situation in OECD countries see Colombo and Tapay (2004).

## Conclusions

We use a static as well as a dynamic panel data analysis to examine the role of economic, socio-demographic, political and institutional country characteristics for three indicators of prepaid health care financing. The first indicator, the share of total prepaid financing as percent of total current health expenditures, measures the relative importance of total prepaid health care financing compared to OOP payments. The second dependent variable is the share of voluntary prepaid financing as percent of total prepaid financing which relates voluntary to compulsory prepaid financing. Finally, we use the share of compulsory health insurance as percent of total compulsory prepaid financing as the third indicator for health care financing and thereby distinguish between compulsory health insurance and tax financed health care.

The dynamic model reveals that the current structure of health care financing is crucially determined by the past. In all of the specifications the lagged dependent variable is significantly positive. Aside the importance of prepaid health care financing in the past we find that voluntary prepaid financing is the lower the higher a country’s GDP/capita is. The ratio of compulsory health insurance over total compulsory prepaid financing increases with increasing GDP/capita. The magnitude of the GDP’s impact does not vary across the quartiles of the GDP/capita distribution. Governmental revenue positively influences the share of prepaid financing and negatively impacts the share of voluntary prepaid financing. Voluntary prepaid financing is rising with increasing development assistance for health but decreases with a country’s regulatory quality and degree of urbanization. The share of compulsory health insurance is higher in countries with a higher agricultural value added and lower regulatory quality. The share of the elderly and the education level only play a minor role in explaining the countries’ health care financing structures. Once we conduct a separate analysis for the rich (high, upper middle income) and poor (lower middle and low income) countries, we find effects similar to those in the main regression, although the importance of the individual determinants sometimes varies between the two subsamples.

Based on the findings in this analysis we argue that more detailed information is needed to adequately capture the individual constraints and decisions related to health care financing. In particular, two variables seems crucial for the individual health care financing decisions: income and health expenditure risks (Gouveia, 1996). Future studies that use individual data, or at least macro indicators which reflect the distribution of income (wealth) and health expenditure risks within a country’s population would allow for a deeper insight into the decisive determinants for the health care financing structures.

## Appendix

See Tables

**Table 4:** Data sources

Database	Link/Reference	Variables used
WHO, Global health expenditure database	https://apps.who.int/nha/database/Select/Indicators/en	*pre_che, vol_pre, hi_com*
World bank, World development indicators	http://databank.worldbank.org/data/source/world-development-indicators	*gdppc_cons, lpr, va_agri, popurb, pop65*
World bank, Worldwide governance indicators project	http://info.worldbank.org/governance/wgi/#home	*regqual*
World bank, Historical classification by income	https://datahelpdesk.worldbank.org/knowledgebase/articles/378834-how-does-the-world-bank-classify-countries	*wbclass*
IMF, World economic outlook database	https://www.imf.org/external/pubs/ft/weo/2015/02/weodata/index.aspx	*govrev*
IMF, Financial development index database	https://data.imf.org/?sk=F8032E80-B36C-43B1-AC26-493C5B1CD33B	*findev*
Institute of health metrics and evaluation	http://ghdx.healthdata.org/record/development-assistance-health-database-1990-2016	*dah*
UN, Human development reports	http://hdr.undp.org/en/data	*myschool*
Center for systemic peace and societal-systems research, Polity IV project, political regime characteristics and Transitions, 1800–2013	http://www.systemicpeace.org/inscrdata.html	*democperm (polity2)*

4 and

**Table 5:** Descriptive statistics

Variable	Obs	Mean	Min	Max	Description
Dependent variables					
pre_che	2303	0.63	0.12	0.97	Total prepaid financing as percent of total current health expenditures
pre_che_lo	2303	0.66	−1.99	3.48	Log-odds ratio of total prepaid financing as percent of current health expenditures
vol_pre	2303	0.17	0.00	0.93	Voluntary prepaid financing as % of total prepaid financing
vol_pre_lo	2226	−1.93	−4.47	2.64	Log-odds ratio of voluntary prepaid financing as % of total prepaid financing
hi_com	2303	0.26	0.00	1.00	Compulsory health insurance as % of compulsory prepaid financing
hi_com_lo	1390	−0.45	−4.37	4.36	Log-odds ratio of compulsory health insurance as % of compulsory prepaid financing
Explanatory variables					
L.lngdppc_q1	2303	1.86	0.00	7.22	Lag of ln(GDP per capita constant)—1st quartile
L.lngdppc_q2	2303	1.83	0.00	8.38	Lag of ln(GDP per capita constant)—2nd quartile
L.lngdppc_q3	2303	2.14	0.00	9.56	Lag of ln(GDP per capita constant)—3rd quartile
L.lngdppc_q4	2303	2.53	0.00	11.63	Lag of ln(GDP per capita constant)—4th quartile
L.govrev	2303	28.84	0.64	72.51	Lag of general government revenue (%)
L.lpr	2303	67.85	37.96	90.34	Lag of labor force participation rate (%) (ILO)
va_agri	2303	12.82	0.03	79.04	Agriculture, value added (% of GDP)
dah_cap	2303	5.63	0.00	184.99	Development assistance for health per capita
findev	2303	0.32	0.03	1.00	Financial development index
popurb	2303	55.97	8.25	100.00	Urban population (%)
pop65	2303	7.66	0.69	26.02	Population ages 65 and above (% of total)
myschool	2303	7.66	1.10	14.10	Mean years of schooling
democperm	2303	0.50	0.00	1.00	Percentage of democratic years (1980–2015)
regqual	2303	−0.01	−2.63	2.26	Regulatory quality
Variables needed for calculation					
cfa_che	2303	52.93	3.00	95.00	Compulsory financing arrangements (CFA) as % of current health expenditure (CHE)
vhi_che	2303	4.58	0.00	50.00	Voluntary health insurance as % of current health expenditure (CHE)
other_che	2303	5.89	0.00	60.00	Other financing arrangements as % of current health expenditure (CHE)
gdppc_cons	2303	12,649.27	194.87	111,968.40	GDP per capita (constant 2005 US$)
govrev	2303	28.99	0.64	72.51	General government revenue (as % of GDP)
lpr	2303	67.89	37.96	90.34	Labor force participation rate, total (% of total population ages 15–64)
dahmio	2303	86.04	0.00	1573.20	Development assistance for health (in mio. constant 2014 USD)
polity2	2286	4.15	−10.00	10.00	Combined polity score
ydemoc	2303	14.49	0.00	36.00	Number of democratic years (1980–2015)

5

## Footnotes

1 For a comprehensive overview of the older literature on the determinants of health care expenditures and financing see Gerdtham & Jönsen (2000).

2 In order to test the sensitivity of the results with respect to the estimation procedure, we use in a robustness check fractions (rather than log-odds ratios) as dependent variables in the static model and follow Papke & Wooldridge (2008) who describe panel data methods for fractional response variables. This robustness check largely support our findings in the base regressions.

3 The final sample comprises 96.8% of the total population and 95.1% of the total current health expenditures of the 189 countries.
